# The bioscience revolution & the biological weapons threat: levers & interventions

**DOI:** 10.1186/1744-8603-5-3

**Published:** 2009-02-16

**Authors:** Mark D'Agostino, Greg Martin

**Affiliations:** 1 Warren Alpert Medical School, Brown University, Providence, Rhode Island, USA; 2 Faculty of Life Science, Genetics Institute, University College London, London, UK

## Abstract

In December 2008, the US Commission on the Prevention of Weapons of Mass Destruction Proliferation and Terrorism, released a report, *World At Risk*. The Report points to the fact that, not only is the use of a weapon of mass destruction in a terrorist attack before the end of 2013, more likely than not, but also to the fact that terrorists are more likely to be able to obtain and use biological weapons than nuclear. This paper examines the recommendations of the report in the context of the historic and geopolitical changes, in particular globalization. The authors highlight the "dual-use" dilemma, as described in the report, as the paradoxical use of technology developed for the benefit of mankind being used for sinister purposes. The mitigation of such a threat lies in broad stakeholder involvement and cooperation, including non-state actors, governments and the bio-tech industry itself. The importance of vigilance measures within the life science community is emphasized and, the authors propose, could include a web-based didactic course in bioterrorism and weapons of mass destruction identification. The site could outline safety protocols, have detailed disaster management tutorials, and could be specifically tailored for different subsets of industry and health professionals. The paper concludes with an endorsement of a multi-pronged approach including strong international guidelines and intelligence cooperation and preparatory measures such as the wide-spread use of detection systems as well as diagnostic decision support systems for bioterrorism detection at the local level.

## Introduction

The US Commission on the Prevention of Weapons of Mass Destruction Proliferation and Terrorism (The Commission), was established in 2007 by the Congress of the United States in House Resolution 1 (P.L. 110-53) to assess and provide a clear and comprehensive strategy and concrete recommendations for prevention activities, initiatives, and programs. In December 2008, the Commission released a report, *World At Risk*, addressing these objectives. The Report's findings received considerable press coverage in the United States and internationally, in part because of the dire prediction that "it is more likely than not that a weapon of mass destruction will be used in a terrorist attack somewhere in the world by the end of 2013", and that "terrorists are more likely to be able to obtain and use a biological weapon than a nuclear weapon" [1]. The bold prediction that an attack with a WMD is likely somewhere in the world by the end of 2013 was arguably not substantiated in the text of the report, potentially fueling the discussion that the biological weapon threat is exaggerated [2]. This issue will not be addressed in this paper. Throughout the Report, many questions were posed that lie squarely at the intersection of globalization and health. In this paper, we aim to address the following questions: Firstly, how will the bioscience revolution change the nature of the biological weapons threat? And which levers and interventions might best mitigate the risk of such an attack?  

## The bioscience revolution

The Commission posed many questions, one of which was *how in the future will the bioscience revolution and the globalization of the biotechnology industry change the nature of the biological weapons threat*? The biotechnology revolution discussed herein began in 1973, 20 years after Watson & Crick's sentinel paper describing the structure of DNA, when Stanley Cohen of Stanford University and Herbert Boyer of the University of California-San Francisco discovered the basic technique for recombinant DNA [3,4]. In the subsequent years, dramatic advances in information technology and processing power helped to spur the rise of many biotechnology companies, principally centered around large research universities within the San Francisco and Boston areas [3]. Presently, thousands of biotechnology companies exist in the United States and throughout the world. This international expansion was driven by a host of factors, such as the growing use of international subcontracting and technological cooperation agreements, including biodefense-related research and vaccine development [5].

## A role for the biotechnology and pharmaceutical industries

The *World at Risk *Report addresses the role of the biotechnology and pharmaceutical industries in addressing the biological weapons threat, and advocates that the United States Department of Health and Human Services (DHHS) press for an international conference of countries with major biotechnology and pharmaceutical industries to discuss the norms and safeguards necessary to keep dangerous pathogens out of the hands of terrorists, and to ensure that the global revolution in the life sciences unfolds safely and securely. Given the vital role that the industries could play in an attack with regards to vaccine dissemination and education, this is a reasonable recommendation; but to have any value the safeguards will require clearly delineated, verifiable safety guidelines with significant sanctions for non-compliance. However, the question of who will regulate the safeguards needs to be addressed. Governmental regulation, through unannounced site visits and investigations would be preferred over self-regulation, particularly if there is a threat of sanctions for non-compliance.

Biotechnology and pharmaceutical companies, governmental agencies (such as the NIH/NSF in the US), and foundations play a critical role in funding life sciences research, particularly in universities. We propose that obtaining grants and continued funding for life sciences research should be made contingent upon proof of adherence to biosafety protocols. This would serve the dual-purpose of increasing the chance of adherence to protocols, while concurrently sensitizing researchers to biosecurity issues. However, this would have little impact inside of the private sector. Working to ensure that the global revolution in the life sciences unfolds safety is a worthwhile goal, but it would likely prove to be extraordinarily difficult to implement given the issues of regulation, enforcement, and the "dual-use" issues arising in biotechnology.

## The dual-use dilemma

The *World at Risk *report addresses the 'dual-use dilemma' of biotechnology, stating that: "at the same time that [biotechnology] has benefited humanity by enabling advances in medicine and agriculture, it has also increased the availability of pathogens and technologies that can be used for sinister purposes" [1]. Emerging technologies and machine automation of complex molecular biological processes has made it easier to synthesize long strands of DNA coding for genes and even entire microbial genomes. By piecing together large fragments of genetic material synthesized in the laboratory, it is possible to assemble highly virulent infectious viruses. At this juncture this process would be technically challenging, expensive, and unreliable. However, as DNA synthesis and manipulation technologies continue to advance at a rapid pace, it will soon be possible to synthesize nearly any virus whose DNA sequence has been decoded [1].

Chyba and Greninger note that experiments performed and published over the last decade-ranging from the incorporation of immune-suppressing interleukin-4 (IL-4) into the mousepox virus to create a deadlier virus able to infect vaccinated animals, to the ability to synthesize viruses from scratch using chemicals on the open market-already demonstrate that the technological know-how to construct dangerous pathogens is widespread [5]. This highlights the dual-use dilemma. Attempting to ban these technologies or stifle technology transfer is not a viable or reasonable option. It is necessary therefore to design policies that can concurrently suppress biological weapons development whilst accommodating and encouraging the spread of dual use technologies for technical and scientific advancement by the life sciences community [6-9].

## The life sciences community

The life sciences community *-defined as universities, medical and veterinary schools, nongovernmental biomedical research institutes, trade associations, and biotechnology and pharmaceutical companies- *has an important role to play in mitigating the bioterrorism threat. The *World at Risk *Report states that the life sciences community must foster a "bottom-up effort to sensitize researchers to biosecurity issues and concerns, and to strive to design and conduct experiments in a way that minimizes safety and security risks" [1]. It is paramount that the life sciences community works toward increased cooperation with intelligence agencies, while concurrently working towards fostering an increased awareness of potential threats at the design phase of experiments. To address the Commission's recommendation of sensitizing researchers to biosecurity issues, we propose the development of a web-based didactic course in bioterrorism and weapons of mass destruction (WMD) identification and treatment for the life sciences community. The site could outline safety protocols, have detailed disaster management tutorials, and could be specifically tailored for different subsets of industry and health professionals including: researchers, medical/veterinary students, graduate students in the biological and life sciences, amongst others. Furthermore, to provide an educational resource for the public, we propose that the biotechnology and/or pharmaceutical industry, as a demonstration of corporate social responsibility, create a pictorial-based patient-centered web interface designed to educate the public and optimize and coordinate use of emergency services in the event of a bioterrorism attack.

## The growing threat of non-state actors

Intelligence estimates appear to agree that the acquisition and dissemination of deadly pathogens would entail fewer hurdles than the theft or production of weapons-grade uranium or plutonium and its assembly into an improvised nuclear device, thus rendering the biological weapons threat greater than the nuclear threat in this respect [1]. With the growing threat of non-state actors obtaining biological weapons and other weapons of mass destruction, over the past five years the international community has launched initiatives to address it. These include the Proliferation Security Initiative (PSI) and UN Security Council Resolution 1540, which were created in 2004 to ensure that states prohibit non-state actors from manufacturing, acquiring or obtaining materials that could support the use of biological or chemical weapons [9,10]. Initiatives have also been launched from the International Committee of the Red Cross (ICRC), the International Criminal Police Organization (Interpol), and the Organization for Economic Cooperation and Development (OECD) [11]. While there are still significant technical hurdles that terrorist groups would need to surpass to be able to weaponize biological agents, synthetic genomics, machine automation of complex molecular biological processes, and a flurry of emerging technologies will make this barrier easier to cross. Furthermore, the Commission found that terrorist groups could upgrade their capabilities by recruiting scientists adept in these technologies. This highlights the importance of fostering a culture of sensitivity to biosecurity issues within the life sciences community, as well as the importance of increasing communication between the life science and intelligence communities.

## Biological weapons convention

The *1972 Convention on the Prohibition of the Development, Production and Stockpiling of Bacteriological (Biological) and Toxin Weapons and on their Destruction *(BWC), entered into force March 26, 1975, has greater than 160 nation-state signatories and provides a multilateral control of the stockpiling and spread of weapons technology. The *World at Risk *report recommends that an action plan for achieving universal adherence to and effective national implementation of the BWC be proposed for adoption at the next review conference in 2011. This is a worthwhile goal, particularly if one believes that those who seek to use biological weapons against a civilian population are likely to obtain the pathogens by stealing them or by recruiting scientists from state-funded programs. However, due to the dual-use nature of biotechnology materials, verification of adherence by a regulatory body or other means remains difficult, if not impossible.

The difficulty is verifying the BWC was highlighted in 2001, when the United States withdrew its support for a draft Biological Weapons Convention (BWC) Protocol, in part because they felt it created the false perception that a biological weapons program could be effectively verified by an international organization [1]. Furthermore, developing nations expressed concern that export controls from the protocol discriminated against them. Since 2003, BWC signatories have held annual expert and political meetings to discuss BWC related domestic legislation, pathogen security, and other issues, and are scheduled to hold review conferences every five years, with the next review conference scheduled for 2011. The continued spread of biotechnology information and materials in the period since the last conference makes it very likely that export controls in developing nations, verification of BWC adherence and the dual-use issue will need to be addressed at the upcoming conference.

## Export controls

Export controls have been employed to mitigate the impact of exports on the development of WMDs with limited success. Examples of such organizations include the Australia Group, an informal forum of countries which, through the harmonization of export controls, seeks to ensure that exports do not contribute to the development of chemical or biological weapons; and the Wassenaar Arrangement on Export Controls for Conventional Arms and Dual-Use Goods and Technologies, signed by 33 nations on July 12, 1996, to suppress the hostile application of dual-use technologies [9]. The Australia Group does not include many of the strategically important developing nations with burgeoning biotechnology industries; and the Wassenaar Arrangement was described by Keller & Nolan as having received scant attention from the policy community and ridicule from the arms lobby because it has "no teeth" [12].

## Levers and interventions

Bioterrorism is a central issue at the interface of globalization and health. This is the result of the globalization of the biotechnology industry, the global spread of biotechnology materials and information, the changing nature of global travel, as well as the ability of infectious diseases to spread worldwide and have a crippling economic impact. Below are the recommendations from the World at Risk Report discussed in this paper, in addition to proposed levers and interventions.

### International Biotechnology Conference

We concur with the Report's recommendation for an international conference of countries with major biotechnology and pharmaceutical industries to discuss the norms and safeguards necessary to keep dangerous pathogens out of the hands of terrorists and to ensure that the global revolution in the life sciences unfolds safely and securely. To be effective, the following should be met:

▪ Safeguards must be clearly delineated and verifiable

▪ Significant sanctions for non-compliance need to be put in place to ensure adherence

▪ Governmental enforcement is preferred over self-regulation

▪ Funding for life sciences research should be made contingent to adherence to safeguards

### Life science community

To address the Report's recommendation that the life sciences community foster a bottom-up effort to sensitize researchers to biosecurity issues and concerns, and to strive to design and conduct experiments in a way that minimizes safety and security risks", the following should be addressed:

▪ Increased cooperation between the life sciences and intelligence communities

▪ Address and minimize bioterrorism threats at the design phase of experiments

▪ Develop a web-based didactic course in bioterrorism and disaster management with detailed safety protocols.

▪ Course should be compulsory for all students and post-doctoral fellows in biological and life science training

▪ Tailor the course for industry and health professionals, medical/veterinary students, and graduate students in the biological and life sciences

▪ Develop pictorial-based patient-centered web interface designed to optimize and coordinate use of emergency services in the event of a bioterrorism attack.

### Policy Interventions

The *World at Risk *report recommends that an action plan for achieving universal adherence to and effective national implementation of the BWC be proposed for adoption at the next review conference in 2011. With regards to the review conference:

▪ The dual-use nature of biotechnology materials will make verification of adherence to safety protocols by a regulatory body or other means critically difficult.

▪ Export controls need to be addressed to ensure that developing nations are not penalized

▪ Immigration and visa policies need to be reevaluated, particularly for students in the life sciences

▪ Strengthen the communication between intelligence agencies and major research universities

## Conclusion

In December 2008, the US Commission on the Prevention of Weapons of Mass Destruction Proliferation and Terrorism released a report, *World At Risk*, reporting that "it is more likely than not that a weapon of mass destruction will be used in a terrorist attack somewhere in the world by the end of 2013", and that "terrorists are more likely to be able to obtain and use a biological weapon than a nuclear weapon." A myriad of economic, technical and political factors are contributing to the elusive and rapidly evolving nature of the biological weapons threat. The dual-use nature of biotechnology and the globalization of the biotechnology industry has made regulation of materials that can be used for biological weapons proliferation cost-prohibitively expensive and laborious. Furthermore, biological weapons programs can be easily concealed, making regulation by an International Atomic Energy Agency (IAEA)-type regulatory body dedicated to biological weapons detection and materials management equally difficult to implement. As a result, a multi-pronged approach must be employed to address the biological weapons threat. Strong international guidelines and intelligence cooperation needs to be complemented by national and local enforcement. Preventative measures and preparatory measures need to be implemented, such as the wide-spread use of detection systems including aerosol sampling, particulate counters and biomass indicators as well as diagnostic decision support systems for bioterrorism detection at the local level [13]. Such a multi-pronged approach is necessary to address this critical national security imperative at the intersection of globalization and health.

## Competing interests

The authors declare that they have no competing interests.

## Authors' contributions

MD drafted most of the text and concepts presented. GM provided text relevant to the global context of the subject matter.
